# Evaluating the Clinical Utility of Routine Sentinel Lymph Node Biopsy and the Value of Adjuvant Chemotherapy in Elderly Patients Diagnosed With Oestrogen Receptor Positive, Clinically Node Negative Breast Cancer

**DOI:** 10.1177/11782234211022203

**Published:** 2021-06-14

**Authors:** Matthew G Davey, Éanna J Ryan, Daniel Burke, Kevin McKevitt, Peter F McAnena, Michael J Kerin, Aoife J Lowery

**Affiliations:** 1 Department of Surgery, Galway University Hospitals, Galway, Republic of Ireland; 2 Discipline of Surgery, Lambe Institute for Translational Research, National University of Ireland Galway, Galway, Republic of Ireland

**Keywords:** Breast cancer, oncogeriatrics, surgical oncology, personalized medicine

## Abstract

**Background:**

Sentinel lymph node biopsy (SLNB) provides staging information and guides adjuvant therapy in early breast cancer (EBC). Routine SLNB in oncogeriatricians with low-risk EBC remains controversial.

**Aims:**

To evaluate axillary management in elderly patients diagnosed with oestrogen receptor positive (ER+), clinically lymph node negative (cLN−) EBC, and to assess whether SLNB affects further axillary management or adjuvant chemotherapy (ACTX) decision making.

**Methods:**

Female patients aged > 65 years, diagnosed with ER+, human epidermal growth factor receptor-2 negative (HER2−), and cLN− breast cancer (BC), who underwent surgery and SLNB were included. Clinicopathological predictors of ACTX and completion axillary lymph node dissection (CALND) were determined. Kaplan-Meier analyses assessed survival outcomes.

**Results:**

A total of 253 patients were included (median age: 72 years, range: 66-90), all underwent SLNB; 50 (19.8%) had lymphatic metastasis on SLNB (SLNB+). Of these, 19 proceeded to CALND (38.0%), 10 (52.6%) of whom had further axillary disease (ALND+). 20 of the 50 SLNB+ patients received ACTX (40.0%) as did 31 of the 203 SLNB− patients (15.2%) (*P* < .001). Oncotype DX (ODX) testing was utilized in 82 cases (32.8%). Younger age (*P* < .001), SLNB+ (*P* < .001) and ODX score (*P* = .003) were all associated with ACTX prescription. ODX > 25 (OR: 4.37, 95% CI: 1.38-13.80, *P* = .012) independently predicted receiving ACTX. Receiving ACTX and proceeding to CALND did not improve disease-free (*P* = .485 and *P* = .345) or overall survival (*P* = .981 and *P* = .646).

**Conclusions:**

Routine SNLB may not be necessary in elderly patients diagnosed with ER+, cLN− EBC. Future oncogeriatric practice is likely to see genomic testing guiding ACTX prescription in this group.

## Introduction

Breast cancer (BC) is the second most common cancer in women worldwide with one-third of BCs occurring in patients above the age of 65.^1^ Historically, surgical management of BC involved extensive resection with radical mastectomy and axillary lymph node dissection (ALND) with considerable associated morbidity.^2^ However, the majority of women now present with early-stage disease (ie, early BC [EBC]), and surgical practice has evolved such that tumour resection via either breast conserving surgery (BCS) or mastectomy with simultaneous sentinel lymph node biopsy (SLNB) for patients with clinically lymph node negative (cLN−) disease is now performed routinely without compromising oncologic outcome.^3^ This paradigm shift towards increasingly conservative surgical approaches to BC has been driven by a desire to minimize morbidity, while maintaining optimal oncologic outcomes.^4^ In the case of the axilla, the development of SLNB facilitated accurate minimally invasive nodal staging to inform therapeutic decision-making based on the presence or absence of axillary metastases.^5^

The accuracy of SLNB as a staging tool was supported by evidence from the National Surgical Adjuvant Bowel and Breast Project (NSABP) B-32 study which demonstrated that patients with a negative SLNB (SLNB−) could be spared ALND.^6^ Further evolution towards conservative axillary management came about following reports from the American College of Surgeons Oncology Group (ACOSOG) Z0011 trial which demonstrated that ALND does not improve survival or local control in BC with less than 3 positive sentinel lymph nodes (LNs).^7,8^ Furthermore, although traditional clinicopathologic features such as tumour size and axillary nodal disease were the primary drivers of adjuvant therapy decision making, the importance of tumour biology has gained increasing relevance in this regard over the last decade. The NSABP B-14, NSABP B-20, Southwest Oncology Group-8814 (or SWOG-8814), and Trial Assigning Individualized Options for Treatment (TAILORx) studies have demonstrated the potential to deliver rationalized adjuvant chemoendocrine prescription based on recurrence scores calculated from genomic assays^5,9-12^ This has led to genomic assays, such as Oncotype DX (ODX) Recurrence Score (RS) (Genomic Health Inc., Redwood City, California) and MammaPrint (Agendia Precision Oncology, Amsterdam, The Netherlands), being incorporated as a component of modern BC management in ER+ cases.

Contemporary BC surgical management has been informed by evidence from well-designed, prospective randomized studies; however, most trials have focused their inclusion criteria on younger participants, possibly due to their greater potential survival benefit as well as their overall suitability for inclusion based on their functional status.^13-15^ This selection bias has led to consistent underrepresentation of elderly patients in clinical trials and a less robust evidence base regarding oncological best practice for these patients. The ‘Choosing Wisely’ campaign was established in 2012 in the United States in an attempt to address this discrepancy in treatment and the associated vulnerability for geriatric patients.^16^ Data published from ‘Choosing Wisely’ suggests a more refined approach to oncogeriatric patient management in those with ER+, HER2−, cLN− BC may be beneficial where possible.^17^ Although routine SLNB has revolutionized surgical management of the axilla for the vast majority of EBC patients, it is not without associated morbidity, and a shift to an even more conservative approach may now be considered for older patients if SLNB as an axillary staging investigation is not providing information that will inform/change further axillary or systemic treatment. This is likely to be the case considering the fact that the primary driver of adjuvant systemic therapy in contemporary BC management is tumour biology.^18,19^

Considering the changes in practice in the US following the Society of Surgical Oncology (SSO) Choosing Wisely recommendations,^17^ the aims of the present study were to

- Evaluate axillary management in an Irish Cohort of elderly patients diagnosed with early stage, ER+ BC;
- Identify if SLNB affects further axillary management or adjuvant systemic therapy decision making in elderly patients diagnosed with ER+ BC.

## Methods

### Patient selection

Local ethical approval was obtained from the Galway University Hospitals Clinical Research Ethics Committee. A single-centre retrospective study was undertaken including all patients above the age of 65 years diagnosed and treated for ER+ EBC in a tertiary referral cancer centre between January 2005 and December 2015. Patients were defined as being elderly if they were ‘aged 66 years or older’ at the time of diagnosis in accordance to the World Health Organization’s definition of ‘elderly’,^20^ and not that of the ‘Choosing Wisely’ campaign. All patients included had a diagnosis of tumour stage 1-2, ER+, HER2−, cLN− BC. Patients were categorized based on age into 3 subgroups; 66-70, 71-75, and >76 years. Patients were identified from a prospectively maintained institutional database and information regarding clinical patient, histopathological tumour, treatment characteristics, and survival data were updated from electronic and medical records.

### Breast cancer diagnosis and work up

All patients underwent triple assessment as part of their EBC workup. Clinical examination was performed by a consultant breast surgeon. Standard radiological assessment consisted of mammography and ultrasound of the breast and ipsilateral axilla. Core biopsy was performed for clinically or radiologically suspicious lesions. All biopsied breast tissue was assessed by a consultant histopathologist with expertise in breast pathology. All breast tissue specimens were analysed in the accredited pathology laboratory at the tertiary referral centre. Staging was performed as per the American Joint Committee on Cancer (AJCC), version 8 Guidelines (2017).^21^

### Histopathological or immunohistochemical tumour evaluation and staging

ER and progesterone receptor (PgR) status were determined in accordance to American Society of Clinical Oncology/College of American Pathology guidelines and reported using Allred scoring.^22-24^ HER2 status was analysed using immunohistochemical analyses,^25^ and fluorescence in-situ hybridization (or FISH) was requested in the case of equivocal 2+ results.^26^ Tumour grade was determined using the Elston Ellis modification of the Scarff-Bloom-Richardson grading system.^27,28^ Lymphatic invasion was assessed using IHC staining with D2-40 and vascular invasion using CD34.^29,30^ Perineural invasion was determined using IHC staining with S-100 and a broad-spectrum keratin stain (AE1/AE3).^31^ MIB1 antibody testing was used to Ki-67 proliferation.^32^

### Multidisciplinary care

All cases were discussed at a BC multidisciplinary meeting attended by consultant breast surgeons, histopathologists, radiologist, medical oncologists, and radiation oncologists. Decisions regarding patient specific treatment are determined from clinical, radiological, and pathological factors, as well as each patient’s performance status in line with international guidelines and best practice. Adjuvant prescription of chemoendocrine therapies for some patients diagnosed with ER+, HER2−, cLN− after 2007 were guided by ODX genomic testing following decisions allowing public reimbursement for the assay in oncological practice by the Irish government.^33^ ODX testing was carried out in Genomic Health Inc, Redwood City, CA, USA.^34^ Neoadjuvant treatment regimens, primary oncological surgeries and adjuvant treatment regimens were carried out in the tertiary referral centre. Patients returned to the tertiary referral centre for examination by a specialized breast surgeon postoperatively and returned for annual clinical and mammographic surveillance after BC treatment.

### Patient follow up and clinical outcomes

Individual patient follow-up and survival data was recorded through a prospectively maintained database. All data was cross-referenced with patient electronic and medical records. Patient mortality was confirmed from data obtained from National Registries. The median and mean lengths of follow-up were assessed using the reverse Kaplan-Meier method.^35^ Definition of DFS was ‘freedom from invasive disease recurrence, a second primary cancer or death’. Patients with ⩾ 3 LNs positive for disease following SLNB, were considered for CALND, as described in the ASOCOG Z0011 study.^7^

### Statistical analysis

Details regarding treatment characteristics within each age group were determined and descriptive statistics were used to inform clinicopathological associations with treatment characteristics (ACTX or CALND). Binary logistic regression analysis was used to ascertain patient and tumour characteristics predicting being in receipt of ACTX or CALND expressed in crude odds ratios (OR) with 95% confidence intervals (CIs). Variables with *P* < .050 in univariable analysis were included in the multivariable logistic regression analysis, which was then used to identify variables that contributed independently to being spared ACTX or CALND. Kaplan-Meier curves and the Log-rank (Mantel-Cox) test were used to associate survival with treatment characteristics. All tests of significance were 2-tailed, with *P* < .050 indicating statistical significance. Data were analysed using Statistical Package for Social Sciences (SPSS) version 26.

## Results

### Patient demographics and clinicopathological characteristics

There were 253 patients aged > 65 years diagnosed with ER+ EBC included in this study. The mean age at diagnosis was 73.2 ± 5.5 years (range: 66-90 years, median age: 72). There were 99 patients aged 66-70 (39.1%), 75 aged 71-75 (29.6%), and 79 patients aged >76 years at diagnosis (31.2%). The mean follow-up was 95.0 months (range: 3.0-185.2 months)

All patients were diagnosed with ER+, HER2−, cLN− primary BC, 121 of which were T1 (47.8%) and 132 of whom had T2 disease (52.2%). The majority had grade 2 tumours (80.6%, 204/253) and 203 had invasive ductal carcinoma histological subtype (80.2%). All tumours were ER+ (100.0%), and the mean ER score was 7.8 ± 0.1 (range: 3-8, median: 8). Overall, 214 tumours had positive PgR expression (84.6%), and the mean PgR score was 5.6 ± 2.1 (range: 0-8, median: 7). Clinicopathological data are outlined in Table 1.

**Table 1.** Clinicopathological, immunohistochemical, and treatment characteristics for 253 patients aged more than 65 diagnosed with oestrogen receptor positive, clinically lymph node negative breast cancer.

Clinicopathological, immunohistochemical and treatment characteristics		
Age at diagnosis (years)	Mean ± SD (range); median	73.2 ± 5.5 (66-90); 72
	Aged 66-70	99 (39.1%)
	Aged 71-75	75 (29.7%)
	Aged > 75	79 (31.2%)
Histological tumour type	Invasive ductal carcinoma	203 (80.2%)
	Invasive lobular carcinoma	42 (16.6%)
	Other (mucinous, micropapillary, papillary, tubular, tubulolobular, etc)	8 (3.2%)
Tumour size (mm)	Mean ± SD (range), median	22.7 ± 10.5 (1-50), 22
Tumour stage	T1	121 (47.8%)
	T2	132 (52.2%)
Tumour grade	1	15 (5.9%)
	2	204 (80.6%)
	3	34 (13.4%)
Lymphovascular invasion, n (%)	Present	43 (17.0%)
	Absent	210 (83.0%)
ER score	Mean ± standard deviation (range), median	7.8 ± 0.1, 8
PgR status PgR score	Positive	214 (84.6%)
	Negative	39 (15.4%)
	Mean ± standard deviation (range), median	5.6 ± 2.1, 7
Ki67 proliferation index (%) (n = 42)	Mean ± standard deviation (range), median	13.6 ± 1.7, 10
Primary surgery	Breast conserving surgery	219 (86.6%)
	Mastectomy	34 (13.4%)
SLNB SLNB nodal yield	Underwent SLNB	253 (100.0%)
	Did not undergo SLNB	0.0 (0.0%)
	Median ± standard deviation (range)	3.0 ± 2.5 (1-12)
OncotypeDX score (n = 82, 32.4%)	Score Mean ± standard deviation (range), median	17.8 ± 9.8 (1-44)
	Low risk (0-10)	13 (15.9%)
	Intermediate risk (11-25)	51 (62.2%)
	High risk (> 25)	18 (22.0%)
Adjuvant endocrine therapy	Yes	253 (100.0%)
	No	0.0 (0.0%)
Adjuvant chemotherapy	Yes	51 (20.2%)
	No	202 (79.8%)
Adjuvant radiotherapy	Yes	173 (68.4%)
	No	80 (31.6%)
Completion ALND	Yes	19 (7.5%)
	No	234 (92.5%)

Abbreviations: ALND, axillary lymph node dissection; ER, oestrogen receptor; PgR, progesterone receptor; SD, standard deviation; SLNB, sentinel lymph node biopsy.

### Treatment characteristics

**Figure 1.**
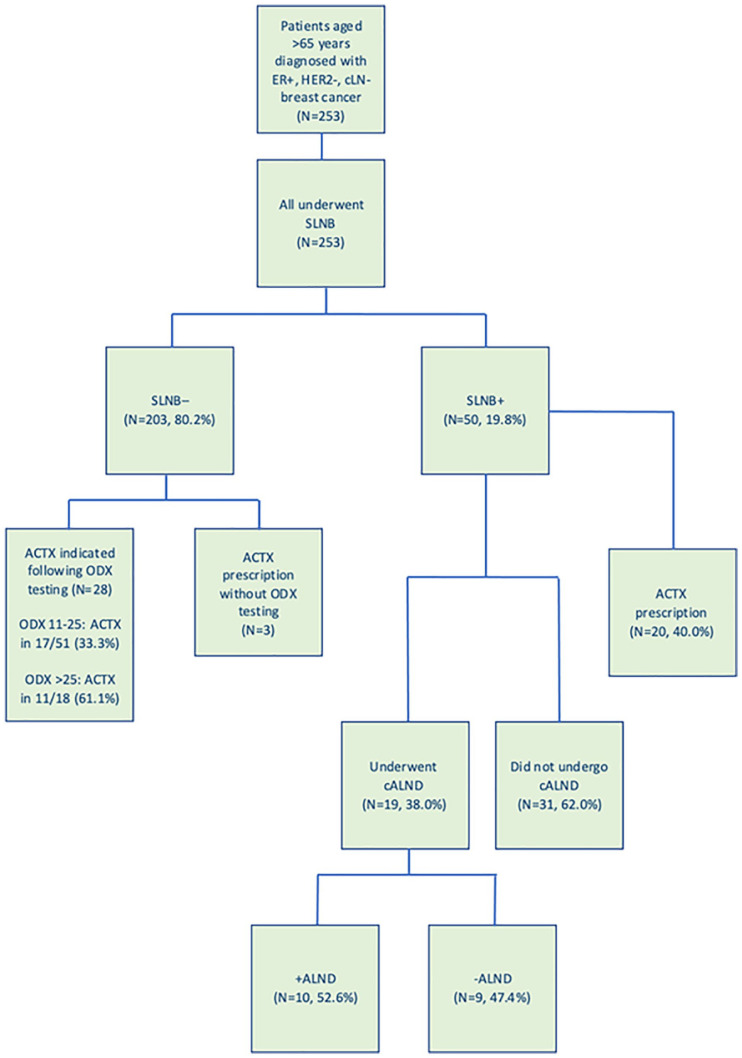
Flow diagram of the clinical utility of sentinel lymph node biopsy in guiding adjuvant treatment strategies in patients diagnosed with oestrogen receptor positive, clinically node negative breast cancer aged greater than 65 years. ACTX indicates adjuvant chemotherapy; cALND, completion axillary lymph node dissection; cLN−, clinically lymph node negative; ER+, oestrogen receptor positive; HER2−, human epidermal growth factor receptor-2 negative; ODX, OncotypeDX Recurrence Score testing; SLNB, sentinel lymph node biopsy.

In total, 219 patients underwent BCS (86.6%). All patients in this series underwent SLNB as part of their oncological staging, with a median of 3.0 ± 2.3 nodes excised for histopathological analysis. Nineteen patients proceeded to CALND (7.5%, 19/253) and rates were similar for all age groups (*P* = .973 χ^2^) (Figure 1). The ODX testing was performed in 82 cases (32.4%, mean score 17.8 ± 8.8, range: 1-44); 13 of which were in the low risk group (ODX < 11, 15.9%), 51 in the intermediate risk group (ODX: 11-25, 62.2%), and 18 in the high-risk group (ODX > 25, 22.0%). All patients received adjuvant EHT (100.0%) and 51 and 173 patients were treated with ACTX and radiotherapy (21.2% and 68.4%, respectively). In patients aged 66-70 years, 31.3% received ACTX (31/99), as did 20.0% aged 71-75 years (15/75), and 6.3% of those aged > 75 years (5/79) (*P* < .001, χ^2^). Treatment characteristics based on age groups and nodal status are outlined in Tables 2 and 3.

**Table 2.** Adjuvant therapy and tumour characteristics for patients with oestrogen receptor positive, clinically node negative breast cancer (N = 253).

Tumour characteristics	Age 66-70 (n = 99, 39.1%)	Age 71-75 (n = 75, 29.7%)	Age > 75 (n = 79, 31.2%)	Total	*P*-value
Grade					.294 χ^2^
1	7 (7.1%)	5 (6.6%)	3 (3.8%)	15 (5.9%)	
2	75 (75.6%)	59 (78.7%)	70 (88.6%)	204 (80.6%)	
3	17 (17.3%)	11 (14.7%)	6 (7.6%)	34 (13.4%)	
Tumour stage					.093†
1	55 (55.6%)	35 (46.7%)	31 (39.8%)	121 (47.8%)	
2	44 (44.4%)	40 (54.3%)	48 (60.2%)	132 (52.2%)	
Histological subtype					.906 χ^2^
IDC	81 (81.8%)	58 (77.3%)	64 (81.0%)	203 (80.2%)	
ILC	15 (15.1%)	15 (20.0%)	12 (15.2%)	42 (16.6%)	
Other	3 (3.0%)	2 (2.7%)	3 (6.3%)	8 (3.2%)	
Oncotype DX Group (n = 82)					.573 χ^2^
Low risk (< 11)	6 (14.0%)	2 (10.0%)	5 (26.3%)	13 (15.9%)	
Intermediate risk (11-25)	28 (65.1%)	12 (60.0%)	11 (57.9%)	51 (62.2%)	
High risk (> 25)	9 (20.9%)	6 (30.0%)	3 (15.8%)	18 (22.0%)	
Surgery					.462†
BCS	84 (38.4%)	68 (31.1%)	67 (30.6%)	219 (86.6%)	
Mastectomy	15 (44.1%)	7 (20.6%)	12 (35.3%)	34 (13.4%)	
Adjuvant chemotherapy					<.001* χ^2^
Underwent treatment	31 (31.3%)	15 (20.0%)	5 (6.3%)	51 (20.2%)	
Did not undergo treatment	68 (68.7%)	60 (80.0%)	74 (93.7%)	202 (79.8%)	
Adjuvant radiotherapy					.184 χ^2^
Underwent treatment	70 (70.7%)	56 (74.7%)	48 (60.8%)	173 (68.4%)	
Did not undergo treatment	29 (28.3%)	19 (25.3%)	31 (39.2%)	80 (31.6%)	
Completion ALND					.973 χ^2^
Underwent treatment	92 (92.9%)	69 (92.0%)	73 (92.4%)	234 (92.5%)	
Did not undergo treatment	7 (7.1%)	6 (8.0%)	6 (7.6%)	19 (7.5%)	

Abbreviations: ALND, axillary lymph node dissection; BCS, breast conserving surgery; IDC, invasive ductal carcinoma; ILC, invasive lobular carcinoma.

χ^2^ denotes chi-square test.

† denotes Fisher’s exact test.

* denotes statistical significance.

**Table 3.** Descriptive statistics illustrating treatment strategies based on nodal status.

Parameter	Node negative on SLNB (N = 203)	Node positive on SLNB (N = 50)	*P*-value
Primary surgery			
BCS	180	38	.063
Mastectomy	23	11	
Adjuvant chemotherapy			
Underwent treatment	31	20	<.001*†
Did not undergo treatment	172	30	
Adjuvant radiotherapy			
Underwent treatment	140	33	.798
Did not undergo treatment	73	17	
Completion ALND			
Underwent treatment	0	19	<.001*†
Did not undergo treatment	203	31	

Abbreviations: ALND, axillary lymph node dissection; BCS, breast conservation surgery; SLNB, sentinel lymph node biopsy.

SLNB; Sentinel lymph node biopsy, BCS; Breast conservation surgery, ALND; axillary lymph node dissection.

† denotes Fisher’s exact test.

* denotes statistical significance.

### Clinical utility of sentinel lymph node biopsy: axillary surgery

Of the 253 patients with cLN− BC, 203 had confirmed node negative disease following SLNB (80.2%) (Figure 1). Fifty patients had histopathological confirmed node positive disease (19.8%), 19 of whom proceeded to CALND (38.0%, 19/50) (*P* < .001†). Of the 234 patients spared CALND, 13.2% had ⩽ 3 positive nodes (N = 31), while all patients who underwent CALND had ⩾ 3 positive nodes at SLNB (100.0%, 19/19). Following CALND, 10 patients had further positive nodes on ALND (ALND+) (10/19, 52.6%).

Using univariable analyses, patients with PgR− tumours (OR: 5.861, 95% CI: 1.138-30.191, *P* = .034) and ⩾ 3 positive LNs on SLNB (OR: 16.200, 95% CI: 1.426-184.057, *P* = .025) were likely to have further positive nodes following CALND. Following multivariable analysis, both PgR− (OR: 5.742, 95% CI: 1.066-30.933, *P* = .042) and ⩾ 3 positive LNs on SLNB (OR: 15.617, 95% CI: 1.169-208.680, *P* = .038) independently predicted patients likely to have ALND+ (Table 4). No patient who proceeded to CALND had previously undergone ODX testing (0/19, 0.0%). For all 253 patients, SLNB+ was associated with proceeding to CALND (*P* < .001†), and there were no other factors predictive of those undergoing CALND (Tables 5 and 6).

**Table 4.** Clinicopathological patient factors predictive of those likely to have further positive lymph nodes following completion axillary lymph node dissection.

Parameter	Univariable			Multivariable		
	OR	95% CI	*P*-value	OR	95% CI	*P*-value
Age < 75	1.104	0.198-6.156	.910			
IDC subtype	1.035	0.118-9.097	.975			
Tumour size > 20 mm	3.055	0.336-27.771	.321			
Grade 3	0.460	0.082-2.588	.378			
LVI	0.998	0.957-1.040	.910			
PgR−	5.861	1.138-30.181	.034^a^	5.742	1.066-30.933	.042*
⩾ 3 +veLN on SLNB	16.200	1.426-184.057	.025^a^	15.617	1.169-208.680	.038*

Abbreviations: CI, confidence interval; IDC, invasive ductal carcinoma; LVI, lymphovascular invasion; OR, odds ratio; PgR−, progesterone receptor negativity; SLNB, sentinel lymph node biopsy; +veLN, positive lymph nodes.

SLNB; sentinel lymph node biopsy.

* denotes statistical significance.

**Table 5.** Clinicopathological characteristics and their relationships with adjuvant chemotherapy prescription and completion axillary lymph node dissection (N = 253).

Clinicopathological characteristics	Received ACTX	Did not receive ACTX	*P*-value	Underwent cALND	Did not undergo cALND	*P*-value
Age						.954†
< 75 years	46	128		7	3	
> 75 years	5	74	<.001* †	8	3	
Histological subtype						.788 χ^2^
IDC	45	158		9	10	
ILC	6	36		1	1	
Other	0	8	.180 χ^2^	0	0	
Tumour stage						.639†
1	21	100		4	4	
2	30	102	.182†	6	7	
Grade						.477 χ^2^
1	0	15		0	0	
2	44	160		7	11	
3	7	27	.257 χ^2^	3	0	
PgR status						.079 χ^2^
PgR+	41	173		6	10	
PgR−	10	29	.386†	4	1	
Ki67 proliferation index (N = 42)						.659†
Ki67 ⩽ 14%	6	26		4	28	
Ki67 > 14%	1	9	.461†	1	9	
Surgery						.308†
BCS	44	175	.551†	8	8	
Mastectomy	7	27		2	3	
SLNB						<.001* †
Positive SLNB	20	30		10	9	
Negative SLNB	31	172	<.001* †	0	0	
Oncotype DX Group (N = 82)						N/A
Low risk (< 11)	2	11	.003* χ^2^	N/A	N/A	
Intermediate risk (11-25)	15	36				
High risk (> 25)	11	7				

Abbreviations: ACTX, adjuvant chemotherapy; BCS, breast conserving surgery; cALND, completion axillary lymph node dissection; IDC, invasive ductal carcinoma; ILC, invasive lobular carcinoma; N/A, not applicable; PgR, progesterone receptor; SLNB, sentinel lymph node biopsy.

χ^2^ denotes chi-square test.

* denotes statistical significance.

† denotes Fisher’s exact test.

**Table 6.** Clinicopathological patient factors predictive of those undergoing completion axillary lymph node dissection.

Parameter	Univariable			Multivariable		
	OR	95% CI	*P*-value	OR	95% CI	*P*-value
Age < 75	0.982	0.359-2.687	.972			
IDC subtype	1.828	0.406-8.229	.432			
Tumour size > 20 mm	1.546	0.559-4.275	.401			
cT2	1.629	0.619-4.282	.323			
Grade 3	1.277	0.357-4.570	.707			
LVI	0.997	0.971-1.024	.842			
PgR−	2.101	0.711-6.211	.179			
Ki67 > 14%	0.778	0.077-7.886	.832			

Abbreviations: CI, confidence interval; cT2, clinical tumour stage 2 disease; IDC, invasive ductal carcinoma; LVI, lymphovascular invasion; OR, odds ratio; PgR−, progesterone receptor negativity.

### Completion axillary lymph node dissection and clinical outcomes

**Figure 2.**
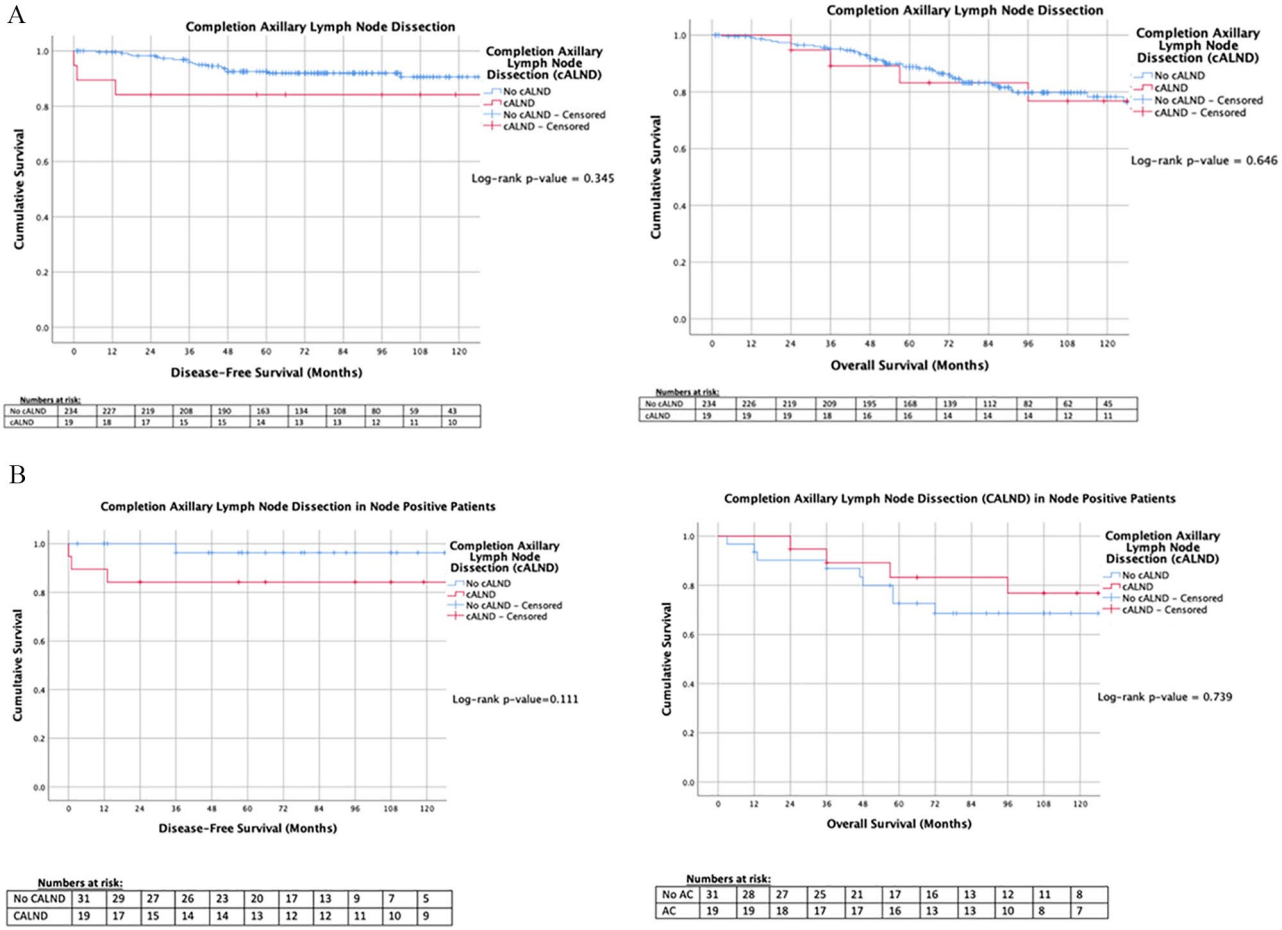
Kaplan-Meier analyses for patients who underwent completion axillary lymph node dissection versus those who did not in the (A) overall cohort, and (B) in those with node positive disease. AC, indicates adjuvant chemotherapy; cALND, completion axillary lymph node dissection.

For all 253 patients, DFS and overall survival (OS) was similar for patients undergoing CALND when compared to those who did not (*P* = .345 and *P* = .646, respectively, log-rank test ‡) (Figure 2A). In patients with LN+ disease, all patients who did not proceed to CALND had ⩽ 3 LN+ (31/31, range: 0-3 LNs, median: 1 ± 1.3 LNs), while 84.2% of those undergoing CALND did had ⩽ 3 LN+ (16/19, range: 1-7, median: 2 ± 2.3 LNs) (*P* = .081 χ^2^). Patients with LN+ disease who were spared CALND had similar LRR, DFS and OS to those who underwent CALND (*P* = .111 and *P* = .739, respectively, ‡) (Figure 2B). Twenty-three patients had disease recurrence, of whom, 73.9% had distant recurrence (17/23), while 26.1% had locoregional recurrence (LRR), (6/23). Of those who developed LRR, 50.0% had previously underwent CALND following SLNB (3/6), while the other 3 were SLN− at the time of primary surgery.

### Clinical utility of sentinel lymph node biopsy: systemic chemotherapy

Overall, 51 patients were treated with ACTX (21.2%), 20 of these were LN+ and 31 were LN− (39.2%, vs 60.8%). ACTX prescription in 28 patients with LN− disease was guided through ODX testing (90.3%, 28/31). Of those undergoing ODX testing and receiving ACTX, the mean ODX score was 22.1 ± 8.1 (range: 11-44, *P* = .452 χ^2^). Figure 2 demonstrates the clinical utility of SLNB in guiding ACTX and CALND.

For the 253 patients included in this series, age at diagnosis, having positive SLNB, and ODX score were all associated with receiving ACTX (*P* < .001†, *P* < .001†, and *P* = .003, respectively, all χ^2^) (Table 5). Using univariable binary logistic regression analyses, it was demonstrated that age less than 75 years at diagnosis (OR: 5.319, 95% CI: 2.024-13.979, *P* = .001), having SLNB+ (OR: 3.699, 95% CI: 1.868-7.323, *P* < .001), and having an ODX score > 25 (OR: 4.345, 95% CI: 1.449-13.026, *P* = .009) were factors predictive of patients receiving ACTX. On multivariable analyses, ODX score > 25 was the sole predictor of patients receiving ACTX (OR: 4.368, 95% CI: 1.382-13.801, *P* = .012) (Table 7).

**Table 7.** Clinicopathological patient factors predictive of those in receipt of adjuvant chemotherapy.

Parameter	Univariable			Multivariable		
	OR	95% CI	*P*-value	OR	95% CI	*P*-value
Age < 75	5.319	2.024-13.979	.001^a^			
IDC subtype	1.709	0.677-4.312	.257			
Tumour size > 20 mm	1.809	0.579-2.048	.791			
cT2	1.401	0.752-2.609	.289			
Grade 3	1.611	0.676-3.838	.282			
LVI	0.997	0.976-1.018	.753			
PgR−	1.455	0.657-3.223	.355			
Ki67 > 14%	0.481	0.051-4.562	.524			
SLNB+	3.699	1.868-7.323	<.001^a^			
ODX score > 25	4.345	1.449-13.026	.009^a^	4.368	1.382-13.801	.012*

Abbreviations: CI, confidence interval; cT2, clinical tumour stage 2 disease; IDC, invasive ductal carcinoma; LVI, lymphovascular invasion; ODX, OncotypeDX Recurrence Score testing; OR, odds ratio; PgR−, progesterone receptor negativity; SLNB+, positive sentinel lymph node biopsy.

OR; odds ratio, 95% CI; 95% confidence interval, IDC; invasive ductal carcinoma, cT2; clinical tumour stage 2 disease, LVI; lymphovascular invasion, PgR-; progesterone receptor negativity,

SLNB+; positive sentinel lymph node biopsy, ODX testing.

* denotes statistical significance.

### Adjuvant chemotherapy and clinical outcomes

**Figure 3.**
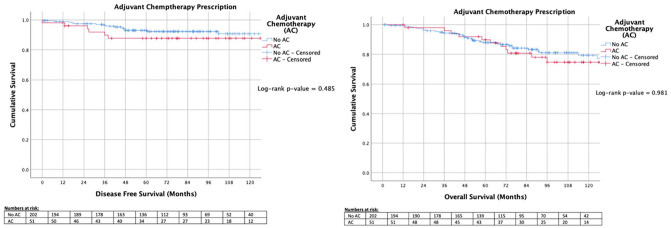
Kaplan-Meier analyses for patients in receipt of adjuvant chemotherapy versus those spared adjuvant chemotherapy. AC indicates adjuvant chemotherapy.

**Figure 4.**
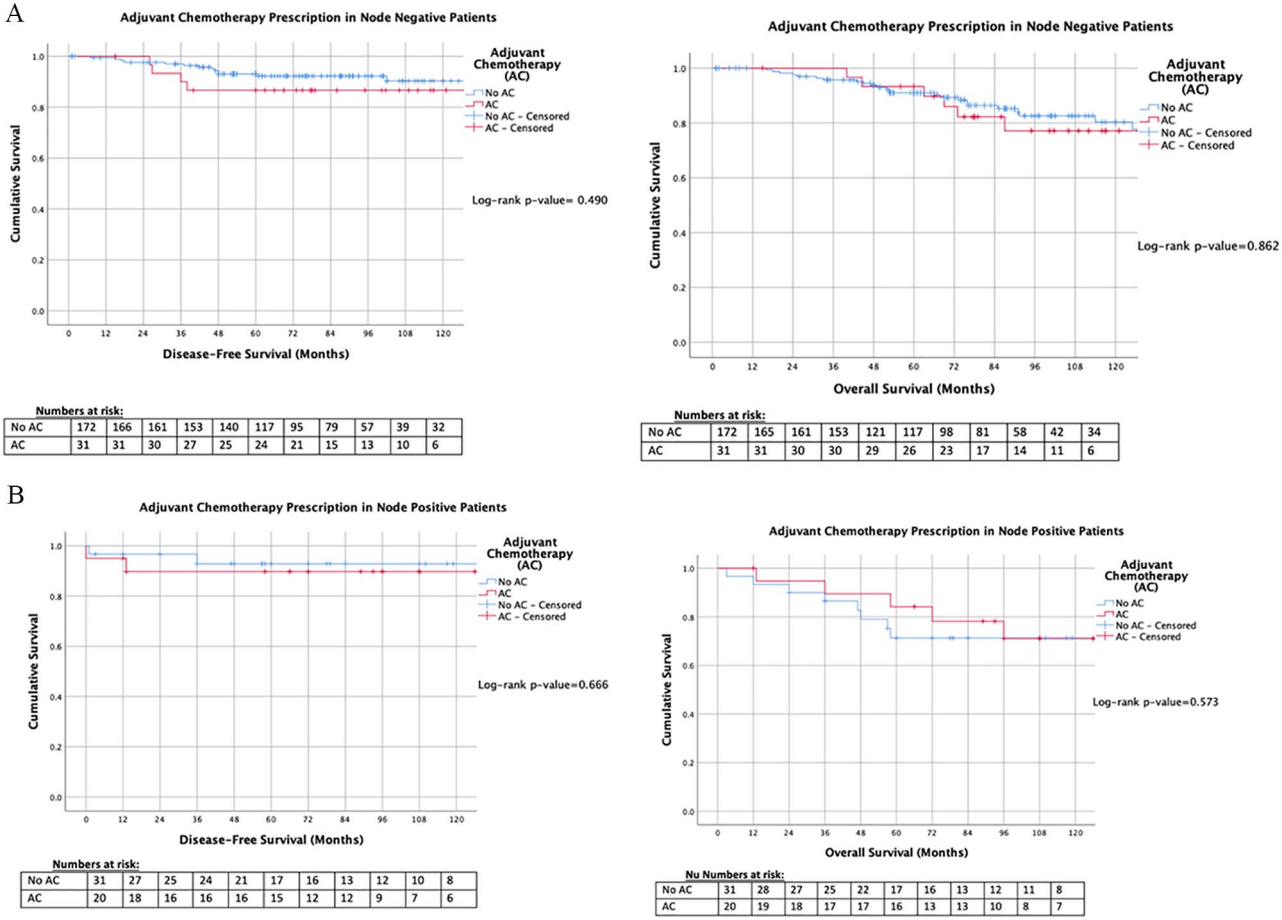
Kaplan-Meier analyses for patients who received adjuvant chemotherapy versus those who did not in the (A) node negative cohort, and (B) in those with node positive disease. AC indicates adjuvant chemotherapy.

Overall, patients in this series demonstrated a DFS of 90.9% (230/253) and an OS of 80.2% (203/253). DFS and OS were similar for patients irrespective of receiving ACTX (*P* = .485 and *P* = .981, respectively, ‡) (Figure 3). For patients with LN− disease, DFS and OS were similar (*P* = .490 and *P* = .862, respectively, ‡) (Figure 4A), and for those with LN+ disease, survival outcomes were similar (*P* = .666 and *P* = .573, respectively, ‡) (Figure 4B).

## Discussion

Traditionally, histopathological features such as tumour size and SLNB+ were predominant factors in guiding decision-making with regard to systemic therapies and the requirement for aggressive locoregional treatment through ALND. However, the majority of elderly patients develop tumours of favourable biology, that is, ER+ve.^36,37^ In the era of personalized medicine, genomic analysis aims to tailor multimodal therapeutic strategies on the basis of case-specific clinicopathological and molecular properties, instead of standard histopathologic and immunohistochemical assessment.^5,9-12,38^ There is also a growing recognition that elderly patients are frequently overlooked as participants in oncological research studies, indirectly leaving this cohort vulnerable to over-investigation and overtreatment. Thus, recent studies have proposed ‘de-escalation’ of local therapies and omission of SLNB in elderly patients with EBC, in particular those with favourable prognosis, such as ER+ disease.^39,40^

The most important finding in this series of elderly patients diagnosed with ER+ EBC is that in spite of SLNB being clinically indicated in all 253 cases, decision making relating to ACTX was more likely to be guided by genomic testing (ODX) than histopathological results of SLNB. Being aged < 75 at diagnosis, having SLNB+, and ODX score > 25 were all factors predictive of ACTX prescription, however, ODX score > 25 was the sole independent predictor following multivariable analysis. These findings are supported by the results of other large volume studies,^41,42^ and are reflected in the modern multimodal treatment paradigm for early ER+ disease, where clinical decision-making regarding tailoring of chemoendocrine therapeutic strategies is primarily determined by genomic tissue analyses.^18,19^ Consequently, the estimated risk of distant recurrence, BC-specific mortality and resulting benefit from ACTX is individualized to the patient and their specific tumour biology, with considerations surrounding functional status evaluated by the multidisciplinary team.

Other results of this univariable analysis are somewhat predictable: Patients aged > 75 years at diagnosis were less likely to undergo ACTX than their counterparts, while those with SLNB+ were likely to be prescribed systemic chemotherapy (Table 7). The loss of physical functionality and resultant disability makes elderly patients less likely to tolerate the toxicities associated with ACTX: data from Given and colleagues suggests that up to 60% of patients aged 65 to 74 years report some form physical disability and unsuitability for systemic therapies.^43^ These results validate the rationale for prudent use of ACTX in the elderly. Histopathological evaluation of the SLNB has traditionally been one of the catalysts for guiding adjuvant systemic therapies, and therefore it is unsurprising that SLNB+ predicted progression to ACTX. With improvements in clinical staging diagnostic modalities, and the increasing availability and affordability of genomic testing, eliminating the routine requirement for invasive axillary sampling in low-risk, elderly BC patients is mooted in modern practice.44

The primary reason for performing SLNB is to allow for accurate staging and to determine the requirement for ACTX and CALND, however survival outcomes following these interventions after SLNB in elderly patients is less well described. In this series, receipt of ACTX failed to improve DFS or OS for elderly patients with ER+ BC, irrespective of nodal status. The authors acknowledge the possibility of a Type II error due to the small study population and few event rates, as is anticipated with EBC, as well as the increase in competing risks for death in older patients. However, this also highlights the challenges faced in optimizing therapy to maximize life expectancy and the quality of life, while minimizing morbidity for this patient group. Data suggests that negative implications of ACTX prescription often outweigh the perceived benefits in elderly and comorbid patients,^45^ with only 15% of patients with cLN− examinations aged > 70 years experienced cancer-related mortality. These statistics demonstrate the difficulty in achieving a survival benefit from prescription of toxic chemotherapy in this group, without risking iatrogenic toxicities or organ failure.^46^ Consequently, the authors propose judicious use of ACTX in elderly patients with EBC, with prescription stratified through individualized risk following genomic analysis, obviating the need for SLNB to guide therapeutic decision-making through axillary staging, in this subset of patients.

As previously described, breakthrough trials have reduced indications for surgical resection through CALND in EBC,^6-8^ and modern studies suggest exemption in elderly patients, given the associated morbidity, minimal survival benefit and oncological control following complete clearance of the axilla.^44,47^ This recent vogue is further supported by randomized data illustrating no added survival advantage from performing CALND in patients aged > 70 years with clinically occult axillary lymph nodes.^48^ Eighty percent of patients in this study had node negative disease, however, for those with node positive disease, proceeding to CALND failed to enhance locoregional control or survival. These results, in tandem with the clinical utility of genomic testing in predicting ACTX prescription, suggest routine SLNB may be unnecessary within this elderly cohort. Focusing on reducing invasive initial diagnostic staging may prove more beneficial in reducing the harmful effects of over-investigation and overtreatment; sonographic axillary staging may be non-inferior to SLNB, with regard to excluding clinically significant metastatic disease in the axilla and requirement for CALND.^49,50^ Thus, less-invasive staging and management of the axilla may reduce morbidity without any compromise in oncologic outcomes for the elderly, with the further possibility of additional cost-savings over current management strategies.^51-53^

This study suffers the inherent limitations of being a single centre, retrospective cohort study implying potential selection, ascertainment and confounding bias. Competing risks affecting survival within the elderly cohort as well as the relatively small numbers powering this study may limit conclusions which can be drawn in relation to survival. Furthermore, the failure to derive clinicopathological predictors of patients undergoing CALND is likely explained by a Type II statistical error due to a limited number of participants proceeding to axillary clearance. However, the data reflects real world practice in a multidisciplinary setting and so the results are relevant to clinicians involved in contemporary BC management.

## Conclusions

Sentinel lymph node biopsy seems less valuable in guiding axillary surgical management and prescription of ACTX than previously perceived in those aged >65 years with ER+ EBC. Genomic testing offers a personalized and less-invasive means of guiding ACTX prescription in this group, and this may better serve this cohort that are at particular risk of over-investigation and treatment. Moreover, CALND and ACTX prescription indicated through SLNB failed to improve survival outcomes for patients in this series, irrespective of nodal status. These findings suggest the omission of SLNB may not be detrimental to clinical outcomes for elderly patients diagnosed with EBC of favourable tumour biology.
